# 1,5-Bis(4-chloro­phen­yl)-3-(2-thien­yl)pentane-1,5-dione

**DOI:** 10.1107/S1600536808038993

**Published:** 2008-11-26

**Authors:** Xianqiang Huang, Feng Xin, Qiu-Lan Shi, Yong Wang, Guo-Dong Wei

**Affiliations:** a Department of Chemistry, Liaocheng University, Liaocheng 252059, People’s Republic of China; b No.4 Middle School of Liaocheng, Liaocheng 252059, People’s Republic of China; c Shandong Donge Experimental High School, Donge, Shandong Province,252200, People’s Republic of China

## Abstract

In the title mol­ecule, C_21_H_16_Cl_2_O_2_S, the five-membered ring is rotationally disordered between two orientations in a 1:1 ratio. In the crystal structure, weak inter­molecular C—H⋯O hydrogen bonds link mol­ecules related by translation along the *a* axis into chains, which are further combined into layers parallel to the *bc* plane *via* C—H⋯π inter­actions. The crystal studied was a racemic twin with a 0.37 (19):0.63 (19) domain ratio.

## Related literature

For the crystal structures of isomers of the title compound, see: Das *et al.* (1994 ▶); Huang *et al.* (2006 ▶). For details of the synthesis, see Bose *et al.* (2004 ▶).
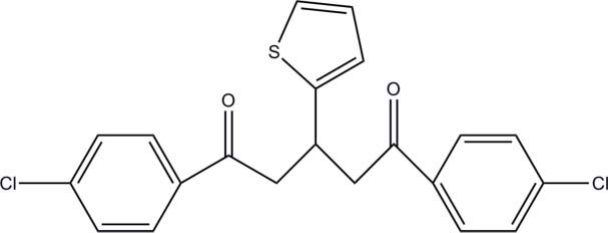


## Experimental

### 

#### Crystal data

C_21_H_16_Cl_2_O_2_S
*M* *_r_* = 403.30
Orthorhombic, 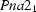

*a* = 7.148 (3) Å
*b* = 14.128 (6) Å
*c* = 19.371 (8) Å
*V* = 1956.3 (14) Å^3^
*Z* = 4
Mo *K*α radiation
μ = 0.45 mm^−1^
*T* = 298 (2) K
0.50 × 0.18 × 0.15 mm

#### Data collection

Bruker SMART CCD area-detector diffractometer
Absorption correction: multi-scan (*SADABS*; Sheldrick, 1996 ▶) *T*_min_ = 0.806, *T*_max_ = 0.935
9549 measured reflections
3430 independent reflections
1466 reflections with *I* > 2σ(*I*)
*R*_int_ = 0.097

#### Refinement

*R*[*F* ^2^ > 2σ(*F* ^2^)] = 0.060
*wR*(*F* ^2^) = 0.248
*S* = 1.01
3430 reflections
274 parameters
93 restraints
H-atom parameters constrained
Δρ_max_ = 0.22 e Å^−3^
Δρ_min_ = −0.24 e Å^−3^
Absolute structure: Flack (1983 ▶); 1650 Friedel pairs
Flack parameter: 0.37 (19)

### 

Data collection: *SMART* (Siemens, 1996 ▶); cell refinement: *SAINT* (Siemens, 1996 ▶); data reduction: *SAINT*; program(s) used to solve structure: *SHELXS97* (Sheldrick, 2008 ▶); program(s) used to refine structure: *SHELXL97* (Sheldrick, 2008 ▶); molecular graphics: *SHELXTL* (Sheldrick, 2008 ▶); software used to prepare material for publication: *SHELXTL*.

## Supplementary Material

Crystal structure: contains datablocks I, global. DOI: 10.1107/S1600536808038993/cv2468sup1.cif

Structure factors: contains datablocks I. DOI: 10.1107/S1600536808038993/cv2468Isup2.hkl

Additional supplementary materials:  crystallographic information; 3D view; checkCIF report

## Figures and Tables

**Table 1:** Hydrogen-bond geometry (Å, °)

*D*—H⋯*A*	*D*—H	H⋯*A*	*D*⋯*A*	*D*—H⋯*A*
C18—H18⋯O2^i^	0.93	2.33	3.175 (12)	150
C10—H10⋯*Cg*^ii^	0.93	2.57	3.489 (10)	171

Symmetry codes: (i) 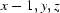; (ii) 
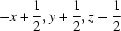
. *Cg* is the centroid of the C16–C21 ring.

**Fig. 1.**
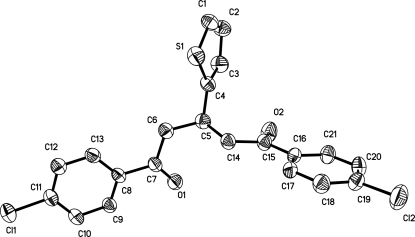
View of (I) showing the atomic labelling and displacement ellipsoids at the 30% level. Only one component of the disordered ring (S1,C1-C4) is shown. Hydrogen atoms are omitted for clarity.

## supplementary crystallographic information

### Comment

In an earlier publication, the "Grindstone Chemistry" method for conducting exothermic reactions in the solvent-free mode has been described (Bose *et al.*, 2004). We tested energy-saving procedures developed in our laboratory for the preparation of 1,5-diketones starting from the fragrant aldehydes and fragrant ketones in the presence of NaOH under solvent-free conditions. Using this method we obtained the title compound, (I).

In (I) (Fig. 1), the bond lengths and angles are normal and correspond to those observed in 1,3,5-triphenyl-pentane-1,5-diketone (Das *et al.*, 1994) and 1,5-diphenyl-3-(2-pyridyl)pentane-1,5-dione (Huang *et al.*, 2006). However, the five-membered ring in (I) is rotationally disordered.

In the crystal, the weak intermolecular C—H···O hydrogen bonds (Table 1) link the molecules related by translations along *a* axis into one-dimensional linear chains, which are further combined into layers parallel to a(b-c) plane *via* C—H···π interactions (Table 1).

### Experimental

4-Chloroacetophenone (6.25 mmol) and thiophene-2-carbaldehyde (3.125 mmol), NaOH (6.25 mmol) were aggregated with glass paddle in an open flask. The resulting mixture was washed with water for several times for removing NaOH, and recrystalized from ethanol, and afforded the title compound as a crystalline solid. Elemental analysis: calculated for C_21_ H_16_ Cl_2_O_2_ S: C 62.54, H 4.00%; Found: C 62.58, H 4.03%.

### Refinement

All H atoms were positioned geometrically and refined using a riding model with C—H = 0.93–0.98 Å and *U*_iso_(H) = *U*_eq_(C). The five-membered ring was treated as disordered between two orientations with nearly equal occupancies refined to 0.501 (11) and 0.499 (11), respectively. The geometries and anisotropic displacement parameters of disordered atoms were refined with soft restraints using the *SHELXL* commands *DFIX*, FLAT and SIMU.

### Crystal data

**Table:**

C_21_H_16_Cl_2_O_2_S	*D*_x_ = 1.369 Mg m^−^^3^
*M**_r_* = 403.30	Mo *K*α radiation λ = 0.71073 Å
Orthorhombic, *P**n**a*2_1_	Cell parameters from 1035 reflections
*a* = 7.148 (3) Å	θ = 2.9–18.1º
*b* = 14.128 (6) Å	µ = 0.45 mm^−^^1^
*c* = 19.371 (8) Å	*T* = 298 (2) K
*V* = 1956.3 (14) Å^3^	Block, colourless
*Z* = 4	0.50 × 0.18 × 0.15 mm
*F*_000_ = 832	

### Data collection

**Table:**

Bruker SMART CCD area-detector diffractometer	3430 independent reflections
Radiation source: fine-focus sealed tube	1466 reflections with *I* > 2σ(*I*)
Monochromator: graphite	*R*_int_ = 0.097
*T* = 298(2) K	θ_max_ = 25.0º
φ and ω scans	θ_min_ = 1.8º
Absorption correction: multi-scan(SADABS; Sheldrick, 1996)	*h* = −8→8
*T*_min_ = 0.806, *T*_max_ = 0.936	*k* = −16→13
9549 measured reflections	*l* = −22→23

### Refinement

**Table:**

Refinement on *F*^2^	Hydrogen site location: inferred from neighbouring sites
Least-squares matrix: full	H-atom parameters constrained
*R*[*F*^2^ > 2σ(*F*^2^)] = 0.060	*w* = 1/[σ^2^(*F*_o_^2^)]
*wR*(*F*^2^) = 0.248	(Δ/σ)_max_ = 0.049
*S* = 1.01	Δρ_max_ = 0.22 e Å^−^^3^
3430 reflections	Δρ_min_ = −0.24 e Å^−^^3^
274 parameters	Extinction correction: SHELXL97 (Sheldrick, 2008), Fc^*^=kFc[1+0.001xFc^2^λ^3^/sin(2θ)]^-1/4^
93 restraints	Extinction coefficient: 0.007 (3)
Primary atom site location: structure-invariant direct methods	Absolute structure: Flack (1983); 1650 Friedel pairs
Secondary atom site location: difference Fourier map	Flack parameter: 0.37 (19)

### Special details

**Table:**

Geometry. All e.s.d.'s (except the e.s.d. in the dihedral angle between two l.s. planes) are estimated using the full covariance matrix. The cell e.s.d.'s are taken into account individually in the estimation of e.s.d.'s in distances, angles and torsion angles; correlations between e.s.d.'s in cell parameters are only used when they are defined by crystal symmetry. An approximate (isotropic) treatment of cell e.s.d.'s is used for estimating e.s.d.'s involving l.s. planes.
Refinement. Refinement of *F*^2^ against ALL reflections. The weighted *R*-factor *wR* and goodness of fit *S* are based on *F*^2^, conventional *R*-factors *R* are based on *F*, with *F* set to zero for negative *F*^2^. The threshold expression of *F*^2^ > σ(*F*^2^) is used only for calculating *R*-factors(gt) *etc*. and is not relevant to the choice of reflections for refinement. *R*-factors based on *F*^2^ are statistically about twice as large as those based on *F*, and *R*- factors based on ALL data will be even larger.

### Fractional atomic coordinates and isotropic or equivalent isotropic displacement parameters (Å^2^)

**Table:**

	*x*	*y*	*z*	*U*_iso_*/*U*_eq_	Occ. (<1)
Cl1	0.2204 (5)	0.35740 (19)	0.37574 (14)	0.0939 (10)	
Cl2	−0.1208 (5)	0.1215 (2)	1.10502 (16)	0.1172 (13)	
O1	0.4870 (9)	0.2902 (4)	0.7007 (3)	0.077 (2)	
O2	0.5943 (10)	0.1389 (5)	0.8854 (4)	0.092 (2)	
S1'	0.4882 (12)	−0.0928 (5)	0.7244 (4)	0.081 (3)	0.501 (11)
C1'	0.670 (3)	−0.1622 (13)	0.7454 (11)	0.072 (6)	0.501 (11)
H1'	0.6698	−0.2277	0.7403	0.086*	0.501 (11)
C2'	0.818 (3)	−0.1125 (15)	0.7705 (12)	0.089 (6)	0.501 (11)
H2'	0.9278	−0.1396	0.7869	0.106*	0.501 (11)
C3'	0.782 (3)	−0.0134 (17)	0.7684 (14)	0.088 (7)	0.501 (11)
H3'	0.8728	0.0324	0.7770	0.106*	0.501 (11)
S1	0.7978 (12)	−0.0198 (6)	0.7932 (5)	0.107 (3)	0.499 (11)
C1	0.772 (3)	−0.1384 (11)	0.7777 (13)	0.085 (6)	0.499 (11)
H1	0.8553	−0.1848	0.7920	0.102*	0.499 (11)
C2	0.609 (4)	−0.1556 (14)	0.7408 (15)	0.096 (7)	0.499 (11)
H2	0.5705	−0.2157	0.7273	0.116*	0.499 (11)
C3	0.510 (4)	−0.0733 (15)	0.7263 (16)	0.097 (7)	0.499 (11)
H3	0.3984	−0.0724	0.7018	0.116*	0.499 (11)
C4	0.5961 (12)	0.0071 (5)	0.7518 (4)	0.059 (2)	
C5	0.5207 (13)	0.1047 (6)	0.7477 (4)	0.058 (2)	
H5	0.6228	0.1486	0.7585	0.070*	
C6	0.4526 (13)	0.1269 (6)	0.6752 (4)	0.060 (2)	
H6A	0.5338	0.0954	0.6424	0.072*	
H6B	0.3279	0.1009	0.6696	0.072*	
C7	0.4467 (13)	0.2307 (6)	0.6581 (5)	0.058 (2)	
C8	0.3898 (11)	0.2589 (6)	0.5866 (4)	0.053 (2)	
C9	0.3827 (13)	0.3539 (6)	0.5708 (4)	0.062 (2)	
H9	0.4142	0.3983	0.6043	0.074*	
C10	0.3292 (14)	0.3844 (7)	0.5056 (5)	0.073 (3)	
H10	0.3216	0.4486	0.4957	0.088*	
C11	0.2880 (14)	0.3186 (7)	0.4564 (4)	0.067 (2)	
C12	0.2986 (13)	0.2235 (6)	0.4700 (5)	0.063 (2)	
H12	0.2735	0.1796	0.4354	0.076*	
C13	0.3468 (12)	0.1938 (6)	0.5352 (5)	0.062 (2)	
H13	0.3506	0.1294	0.5451	0.074*	
C14	0.3630 (13)	0.1219 (6)	0.7999 (4)	0.060 (2)	
H14A	0.2971	0.1793	0.7872	0.072*	
H14B	0.2748	0.0699	0.7972	0.072*	
C15	0.4286 (14)	0.1311 (6)	0.8727 (5)	0.064 (2)	
C16	0.2903 (16)	0.1309 (5)	0.9295 (4)	0.057 (2)	
C17	0.1016 (14)	0.1270 (6)	0.9166 (5)	0.061 (2)	
H17	0.0591	0.1252	0.8713	0.073*	
C18	−0.0272 (14)	0.1257 (7)	0.9706 (5)	0.077 (3)	
H18	−0.1550	0.1233	0.9616	0.092*	
C19	0.0376 (16)	0.1278 (6)	1.0372 (5)	0.071 (3)	
C20	0.2218 (17)	0.1315 (7)	1.0516 (5)	0.080 (3)	
H20	0.2633	0.1324	1.0971	0.096*	
C21	0.3488 (14)	0.1341 (7)	0.9974 (4)	0.071 (3)	
H21	0.4760	0.1379	1.0070	0.085*	

### Atomic displacement parameters (Å^2^)

**Table:**

	*U*^11^	*U*^22^	*U*^33^	*U*^12^	*U*^13^	*U*^23^
Cl1	0.119 (2)	0.0946 (19)	0.0679 (17)	0.0109 (16)	−0.0102 (16)	0.0201 (14)
Cl2	0.114 (3)	0.163 (3)	0.075 (2)	0.021 (2)	0.0267 (17)	−0.0001 (17)
O1	0.106 (6)	0.060 (4)	0.063 (4)	−0.004 (4)	−0.015 (4)	−0.002 (3)
O2	0.063 (5)	0.147 (6)	0.067 (5)	−0.005 (4)	−0.010 (4)	−0.016 (4)
S1'	0.099 (6)	0.075 (5)	0.070 (4)	−0.012 (4)	0.003 (4)	0.007 (3)
C1'	0.091 (12)	0.063 (10)	0.062 (10)	0.013 (9)	0.010 (10)	0.018 (8)
C2'	0.106 (13)	0.077 (11)	0.083 (11)	−0.001 (10)	−0.002 (10)	0.011 (10)
C3'	0.101 (13)	0.081 (12)	0.083 (11)	0.012 (11)	−0.013 (11)	0.009 (10)
S1	0.092 (5)	0.084 (5)	0.146 (8)	0.026 (3)	−0.034 (5)	−0.004 (4)
C1	0.086 (12)	0.048 (10)	0.122 (13)	0.014 (10)	0.005 (11)	0.013 (9)
C2	0.099 (12)	0.072 (11)	0.118 (13)	0.019 (11)	−0.001 (11)	0.016 (10)
C3	0.093 (13)	0.063 (12)	0.135 (14)	0.018 (11)	−0.003 (12)	0.013 (11)
C4	0.056 (6)	0.067 (6)	0.054 (5)	0.000 (5)	−0.006 (4)	0.010 (4)
C5	0.051 (6)	0.061 (5)	0.063 (6)	0.000 (4)	−0.004 (4)	0.003 (4)
C6	0.057 (6)	0.064 (6)	0.058 (6)	−0.002 (4)	0.005 (4)	0.011 (4)
C7	0.054 (6)	0.067 (6)	0.055 (5)	0.009 (5)	0.003 (4)	0.016 (5)
C8	0.044 (5)	0.060 (6)	0.056 (5)	0.006 (4)	0.000 (4)	0.010 (5)
C9	0.065 (6)	0.059 (6)	0.062 (6)	0.000 (5)	−0.016 (5)	−0.001 (4)
C10	0.081 (8)	0.069 (6)	0.070 (7)	0.004 (5)	−0.009 (6)	0.016 (5)
C11	0.076 (7)	0.069 (6)	0.057 (6)	−0.002 (5)	−0.001 (5)	0.015 (5)
C12	0.057 (6)	0.071 (6)	0.062 (6)	0.001 (5)	0.004 (4)	0.005 (5)
C13	0.061 (7)	0.061 (6)	0.064 (6)	0.010 (4)	0.004 (4)	0.006 (5)
C14	0.060 (6)	0.070 (6)	0.051 (5)	0.001 (4)	−0.008 (4)	0.004 (4)
C15	0.059 (6)	0.082 (6)	0.050 (5)	0.003 (5)	−0.009 (5)	−0.005 (4)
C16	0.074 (7)	0.049 (5)	0.047 (5)	0.002 (5)	−0.007 (5)	−0.007 (4)
C17	0.068 (7)	0.067 (6)	0.048 (5)	0.013 (5)	−0.003 (5)	0.002 (4)
C18	0.056 (7)	0.106 (8)	0.068 (7)	0.009 (5)	−0.004 (6)	0.002 (5)
C19	0.092 (9)	0.064 (6)	0.058 (6)	0.001 (5)	0.005 (6)	−0.015 (4)
C20	0.090 (9)	0.106 (8)	0.044 (5)	−0.015 (6)	−0.009 (6)	0.003 (5)
C21	0.070 (7)	0.090 (7)	0.053 (6)	−0.007 (5)	−0.013 (5)	−0.009 (4)

### Geometric parameters (Å, °)

**Table:**

Cl1—C11	1.724 (9)	C6—H6B	0.9700
Cl2—C19	1.736 (11)	C7—C8	1.498 (11)
O1—C7	1.213 (10)	C8—C9	1.377 (10)
O2—C15	1.215 (10)	C8—C13	1.389 (11)
S1'—C1'	1.678 (16)	C9—C10	1.387 (12)
S1'—C4	1.694 (10)	C9—H9	0.9300
C1'—C2'	1.359 (17)	C10—C11	1.364 (13)
C1'—H1'	0.9300	C10—H10	0.9300
C2'—C3'	1.423 (18)	C11—C12	1.371 (12)
C2'—H2'	0.9300	C12—C13	1.376 (12)
C3'—C4	1.401 (18)	C12—H12	0.9300
C3'—H3'	0.9300	C13—H13	0.9300
S1—C4	1.693 (10)	C14—C15	1.490 (12)
S1—C1	1.713 (16)	C14—H14A	0.9700
C1—C2	1.385 (18)	C14—H14B	0.9700
C1—H1	0.9300	C15—C16	1.479 (13)
C2—C3	1.390 (18)	C16—C17	1.373 (12)
C2—H2	0.9300	C16—C21	1.381 (12)
C3—C4	1.383 (19)	C17—C18	1.393 (13)
C3—H3	0.9300	C17—H17	0.9300
C4—C5	1.482 (10)	C18—C19	1.372 (14)
C5—C6	1.518 (11)	C18—H18	0.9300
C5—C14	1.535 (13)	C19—C20	1.347 (14)
C5—H5	0.9800	C20—C21	1.389 (14)
C6—C7	1.504 (10)	C20—H20	0.9300
C6—H6A	0.9700	C21—H21	0.9300
			
C1'—S1'—C4	93.3 (9)	C9—C8—C13	118.6 (7)
C2'—C1'—S1'	112.8 (13)	C9—C8—C7	118.3 (7)
C2'—C1'—H1'	123.6	C13—C8—C7	123.1 (7)
S1'—C1'—H1'	123.6	C8—C9—C10	120.9 (8)
C1'—C2'—C3'	111.1 (17)	C8—C9—H9	119.5
C1'—C2'—H2'	124.5	C10—C9—H9	119.5
C3'—C2'—H2'	124.4	C11—C10—C9	119.0 (9)
C4—C3'—C2'	112.3 (17)	C11—C10—H10	120.5
C4—C3'—H3'	123.8	C9—C10—H10	120.5
C2'—C3'—H3'	123.8	C10—C11—C12	121.4 (8)
C4—S1—C1	92.5 (9)	C10—C11—Cl1	118.6 (7)
C2—C1—S1	110.7 (13)	C12—C11—Cl1	120.1 (7)
C2—C1—H1	124.6	C11—C12—C13	119.3 (9)
S1—C1—H1	124.6	C11—C12—H12	120.4
C1—C2—C3	112.6 (17)	C13—C12—H12	120.4
C1—C2—H2	123.7	C12—C13—C8	120.7 (8)
C3—C2—H2	123.7	C12—C13—H13	119.6
C4—C3—C2	112.9 (18)	C8—C13—H13	119.6
C4—C3—H3	123.5	C15—C14—C5	114.0 (8)
C2—C3—H3	123.5	C15—C14—H14A	108.8
C3—C4—C3'	109.4 (15)	C5—C14—H14A	108.8
C3—C4—C5	125.7 (13)	C15—C14—H14B	108.8
C3'—C4—C5	123.5 (12)	C5—C14—H14B	108.8
C3—C4—S1	111.3 (12)	H14A—C14—H14B	107.7
C3'—C4—S1	15.2 (13)	O2—C15—C16	120.0 (9)
C5—C4—S1	123.0 (7)	O2—C15—C14	120.4 (9)
C3—C4—S1'	2.8 (15)	C16—C15—C14	119.6 (9)
C3'—C4—S1'	109.4 (11)	C17—C16—C21	118.1 (9)
C5—C4—S1'	126.3 (7)	C17—C16—C15	121.4 (8)
S1—C4—S1'	110.4 (6)	C21—C16—C15	120.4 (9)
C4—C5—C6	111.1 (7)	C16—C17—C18	120.9 (8)
C4—C5—C14	112.3 (7)	C16—C17—H17	119.5
C6—C5—C14	109.9 (7)	C18—C17—H17	119.5
C4—C5—H5	107.8	C19—C18—C17	118.9 (9)
C6—C5—H5	107.8	C19—C18—H18	120.6
C14—C5—H5	107.8	C17—C18—H18	120.6
C7—C6—C5	114.5 (7)	C20—C19—C18	121.7 (10)
C7—C6—H6A	108.6	C20—C19—Cl2	118.9 (8)
C5—C6—H6A	108.6	C18—C19—Cl2	119.4 (9)
C7—C6—H6B	108.6	C19—C20—C21	118.9 (9)
C5—C6—H6B	108.6	C19—C20—H20	120.6
H6A—C6—H6B	107.6	C21—C20—H20	120.6
O1—C7—C8	120.7 (8)	C16—C21—C20	121.5 (10)
O1—C7—C6	121.2 (8)	C16—C21—H21	119.3
C8—C7—C6	118.1 (8)	C20—C21—H21	119.3
			
C4—S1'—C1'—C2'	−2.6 (7)	C5—C6—C7—C8	177.1 (8)
S1'—C1'—C2'—C3'	−3.5 (9)	O1—C7—C8—C9	−0.6 (13)
C1'—C2'—C3'—C4	9.7 (17)	C6—C7—C8—C9	179.5 (8)
C4—S1—C1—C2	0.2 (7)	O1—C7—C8—C13	178.3 (9)
S1—C1—C2—C3	−0.1 (10)	C6—C7—C8—C13	−1.5 (12)
C1—C2—C3—C4	−0.1 (19)	C13—C8—C9—C10	1.6 (14)
C2—C3—C4—C3'	16 (3)	C7—C8—C9—C10	−179.4 (9)
C2—C3—C4—C5	−176.9 (13)	C8—C9—C10—C11	−1.7 (16)
C2—C3—C4—S1	0(2)	C9—C10—C11—C12	0.0 (16)
C2—C3—C4—S1'	−73 (26)	C9—C10—C11—Cl1	179.6 (8)
C2'—C3'—C4—C3	−14 (2)	C10—C11—C12—C13	1.8 (14)
C2'—C3'—C4—C5	178.6 (12)	Cl1—C11—C12—C13	−177.8 (8)
C2'—C3'—C4—S1	85 (5)	C11—C12—C13—C8	−1.9 (14)
C2'—C3'—C4—S1'	−11.3 (19)	C9—C8—C13—C12	0.2 (13)
C1—S1—C4—C3	−0.3 (15)	C7—C8—C13—C12	−178.8 (9)
C1—S1—C4—C3'	−86 (4)	C4—C5—C14—C15	73.6 (9)
C1—S1—C4—C5	176.9 (11)	C6—C5—C14—C15	−162.3 (7)
C1—S1—C4—S1'	2.5 (10)	C5—C14—C15—O2	11.0 (12)
C1'—S1'—C4—C3	100 (27)	C5—C14—C15—C16	−169.3 (7)
C1'—S1'—C4—C3'	7.9 (13)	O2—C15—C16—C17	176.7 (8)
C1'—S1'—C4—C5	177.6 (10)	C14—C15—C16—C17	−3.1 (12)
C1'—S1'—C4—S1	−8.2 (9)	O2—C15—C16—C21	−3.8 (13)
C3—C4—C5—C6	−47.4 (19)	C14—C15—C16—C21	176.4 (8)
C3'—C4—C5—C6	117.6 (16)	C21—C16—C17—C18	−0.4 (12)
S1—C4—C5—C6	135.8 (8)	C15—C16—C17—C18	179.1 (8)
S1'—C4—C5—C6	−50.7 (11)	C16—C17—C18—C19	−0.3 (13)
C3—C4—C5—C14	76.2 (18)	C17—C18—C19—C20	0.2 (14)
C3'—C4—C5—C14	−118.8 (15)	C17—C18—C19—Cl2	−177.5 (7)
S1—C4—C5—C14	−100.6 (9)	C18—C19—C20—C21	0.6 (14)
S1'—C4—C5—C14	72.9 (10)	Cl2—C19—C20—C21	178.3 (7)
C4—C5—C6—C7	−156.7 (8)	C17—C16—C21—C20	1.2 (13)
C14—C5—C6—C7	78.5 (10)	C15—C16—C21—C20	−178.3 (8)
C5—C6—C7—O1	−2.7 (13)	C19—C20—C21—C16	−1.3 (14)

### Hydrogen-bond geometry (Å, °)

**Table:**

*D*—H···*A*	*D*—H	H···*A*	*D*···*A*	*D*—H···*A*
C18—H18···O2^i^	0.93	2.33	3.175 (12)	150
C10—H10···Cg^ii^	0.93	2.57	3.489 (10)	171

Symmetry codes: (i) *x*−1, *y*, *z*; (ii) −*x*+1/2, *y*+1/2, *z*−1/2.
